# Recommendations for good practice in MS-based lipidomics

**DOI:** 10.1016/j.jlr.2021.100138

**Published:** 2021-10-16

**Authors:** Harald C. Köfeler, Robert Ahrends, Erin S. Baker, Kim Ekroos, Xianlin Han, Nils Hoffmann, Michal Holčapek, Markus R. Wenk, Gerhard Liebisch

**Affiliations:** 1 Core Facility Mass Spectrometry, Medical University of Graz, Graz, Austria; 2 Department for Analytical Chemistry, University of Vienna, Vienna, Austria; 3 Department of Chemistry, North Carolina State University, Raleigh, NC, USA; 4 Lipidomics Consulting Ltd., Esbo, Finland; 5 Barshop Inst Longev & Aging Studies, Univ Texas Hlth Sci Ctr San Antonio, San Antonio, TX, USA; 6 Center for Biotechnology, Universität Bielefeld, Bielefeld, Germany; 7 Department of Analytical Chemistry, Faculty of Chemical Technology, University of Pardubice, Pardubice, Czech Republic; 8 Singapore Lipidomics Incubator (SLING), Department of Biochemistry, YLL School of Medicine, National University of Singapore, Singapore, Singapore; 9 Institute of Clinical Chemistry and Laboratory Medicine, Regensburg University Hospital, Regensburg, Germany

**Keywords:** lipidomics, metabolomics, MS, chromatography, ion mobility spectrometry, phospholipids, sphingolipids, LC-MS, lipid identification, DTIMS, drift tube ion mobility spectrometry, FAIMS, field asymmetric waveform ion mobility spectrometry, HILIC, hydrophilic interaction liquid chromatography, HRMS, high-resolution MS, ICR, ion cyclotron resonance, ILS, International Lipidomics Society, IMS, ion mobility spectrometry, IS, internal standard, LCF, lipid class-specific fragment, LPA, lysophosphatidic acid, LSI, Lipidomics Standards Initiative, PC, phosphatidylcholine, QqQ, triple quadrupole, QTOF, quadrupole TOF, RP, reverse-phase, RT, retention time, S1P, sphingosine-1-phosphate, SPE, solid phase extraction, SRM, selected reaction monitoring, TG, triglyceride, TIMS, trapped ion mobility spectrometry, TWIMS, traveling wave ion mobility spectrometry, UHPLC, ultrahigh-performance liquid chromatography, UHPSFC, ultrahigh-performance supercritical fluid chromatography

## Abstract

In the last 2 decades, lipidomics has become one of the fastest expanding scientific disciplines in biomedical research. With an increasing number of new research groups to the field, it is even more important to design guidelines for assuring high standards of data quality. The Lipidomics Standards Initiative is a community-based endeavor for the coordination of development of these best practice guidelines in lipidomics and is embedded within the International Lipidomics Society. It is the intention of this review to highlight the most quality-relevant aspects of the lipidomics workflow, including preanalytics, sample preparation, MS, and lipid species identification and quantitation. Furthermore, this review just does not only highlights examples of best practice but also sheds light on strengths, drawbacks, and pitfalls in the lipidomic analysis workflow. While this review is neither designed to be a step-by-step protocol by itself nor dedicated to a specific application of lipidomics, it should nevertheless provide the interested reader with links and original publications to obtain a comprehensive overview concerning the state-of-the-art practices in the field.

Lipidomics is the science based on large-scale lipid determination by bioanalytical methods, including the interpretation of these data in a broader biological context. Because of a combination of high sensitivity and specificity, MS is the method of choice (1, 2). This review article focuses on ESI because according to Web of Science, more than two-thirds of the lipidomics publications since 2010 used this ionization technique. The other two ionization techniques worth mentioning are MALDI and electron impact/chemical ionization in combination with GC-MS. Since MALDI is overwhelmingly used for MS imaging, which is beyond the scope of this article, and GC-MS does not provide comprehensive lipidomic data but only lipid classes like fatty acyl and sterols, these two ionization techniques are not covered in this review. When coupled to separation techniques like chromatography and ion mobility, ESI-based mass spectrometric analysis can even increase its analytical performance. While in the very beginning, only small sets of lipids were determined by GC-MS, the invention of ESI enabled the acquisition of many lipid classes in one analytical run (3). This development led from overwhelmingly direct infusion MS techniques in the mid-90s (4) to the emerging of a novel discipline called lipidomics from the early new millennium onward (5, 6). Nowadays, the analytical bases of this science are coarsely divided into direct infusion (shotgun) lipidomics (7) and separation coupled lipidomics (8, 9). It has been recognized within the last decade that the steps after data acquisition are crucial for the quality of reported studies: software-assisted data processing, including not only identification and quantitation of lipids but also further biological interpretation of the resulting large datasets (10) and integration with other omics data (11). It has further been recognized in the last decade that the field desperately needs some sort of quality assurance. The first step was the design of a shorthand nomenclature for lipids, which reflects the experimental evidence for the existence of a lipid (12, 13). The take-home message conveyed by this group of authors was: “Report only what is experimentally proven, and clearly state where assumptions were made.” Within the following years, this project evolved into the Lipidomics Standards Initiative (LSI) (https://lipidomics-standards-initiative.org/), a platform concerned by coordination of quality issues in the lipidomics field (10). But since the quality issues within the field soon expanded beyond nomenclature aspects and data acquisition, some core members of the LSI group decided to found a scientific society, the International Lipidomics Society (ILS) (https://lipidomicssociety.org/). Having ILS as scientific society is not only beneficial for developing analytical quality standards but also covers other aspects of interest for the community. Divided into several interest groups, ILS coordinates ring trials in clinical lipidomics, the availability of reference materials, ontology issues, ring trials in bioinformatics, and it is the interface between the community and instrument developers (vendors). But above all, ILS is the communication platform needed by the community, which connects scientists by organizing conferences (International Lipidomics Conference), virtual seminar series, and so on.

This review article aims at giving an overview about the state of the art in the lipidomics field with particular emphasis on recommended best practice examples guiding newcomers and less experienced researchers in the field. Therefore, it will include all aspects of the lipidomics workflow from preanalytics to data analysis, as depicted in Fig. 1.

**Fig. 1:**
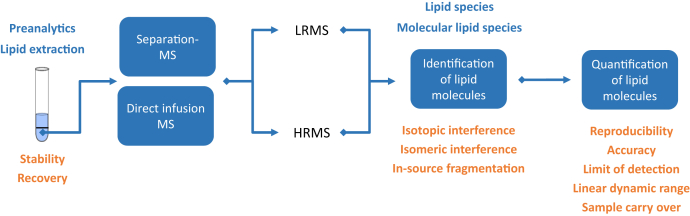
Lipidomic workflow. Typical lipidomic workflows including lipid extraction, direct infusion, or separation-based mass spectrometric analysis by nominal resolution, that is, low-resolution MS or HRMS. Data analysis includes lipid identification at respective structure level, for example, lipid species or lipid molecular species level and their quantification. Important considerations to achieve high-quality results are depicted in orange and are discussed in the respective sections.

## Preanalytics and sample preparation

The first and clearly a critical step in any lipidomics workflow is sampling and subsequent sample processing. Especially, tissues should always be immediately frozen in liquid nitrogen, whereas biofluids like plasma should be either immediately processed or frozen at −80°C. If samples are kept for too long at room temperature, enzymatic and chemical degradation processes could result in lipid peroxidation or hydrolysis (14, 15, 16). It was even observed that samples, which were kept at room temperature and pH >6 for longer periods, showed scrambling of fatty acyls in lysophospholipids, resulting in the loss of regioisomeric specificity (17). Inappropriate preanalytic conditions may change the concentration dramatically like for lysolipids, lysophosphatidic acid (LPA) and sphingosine-1-phosphate (S1P), which are generated instantly after drawing blood samples (15) and therefore require special precautions to preserve in vivo concentrations (18). Lipolytic activity (19), which may continue even after the addition of organic solvents like alcohol and could be monitored by lipid class ratios reflecting degradation (20), is highly undesirable in lipidomics. Generally, the analysis of potential degradation products like oxidized lipids, lysophospholipids, or phosphatidic acid needs a particular precaution to ensure that they do not just arise artificially from sample processing. If possible, it is always good advice to process samples immediately. If unprocessed or processed samples are stored at −80°C for a longer period, then the stability should be verified for the studied lipid species.

**Fig. 2:**
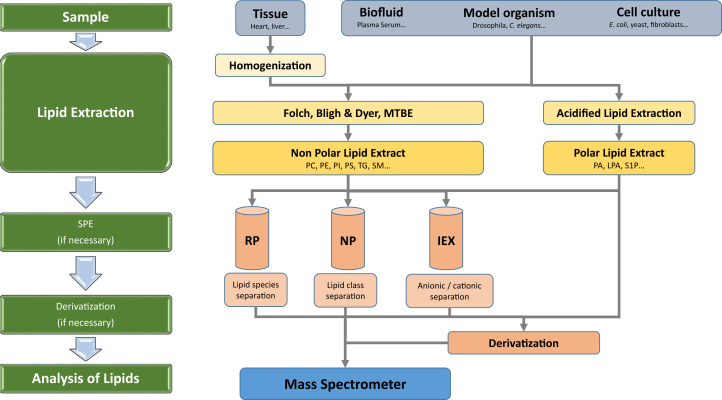
Modular sample preparation workflow. Various sample types (powder blue) are extracted by liquid-liquid extraction (yellow) and eventually enriched by SPE or derivatized (reddish) before they are subjected to mass spectrometric analysis (blue).

Liquid-liquid extraction is the gold standard in lipidomic sample preparation. The historically most widely used methods rely on biphasic chloroform-methanol-water mixtures, such as the methods described by Lebaron and Folch (21) and Bligh and Dyer (22) (Fig. 2). While the Bligh and Dyer protocol has a higher proportion of methanol and is thus slightly more suitable for polar lipids (e.g., glycerophospholipids), the Folch protocol has a higher content of nonpolar chloroform in its organic phase, which increases the solubility of nonpolar lipids (e.g., triglyceride [TG]). Within the last decade, the supremacy of these two classical methods was challenged by a new liquid-liquid extraction method based on methyl-*tert*-butyl ether for reasons of reduced toxicity and improved sample handling (23). When polar anionic lipids, such as LPA or S1P, are of interest, an acidified Bligh and Dyer protocol should be the extraction method of choice (24). Using this extraction, it is of utmost importance to strictly keep to the protocol concerning the concentration of HCl and the extraction time, because otherwise acid-sensitive hydrolysis effects could cause significant alternations of natural concentrations of these sensitive lipid classes. Particularly for large-scale lipidomics studies, biphasic liquid-liquid extraction protocols are often replaced by monophasic methods based on the protein precipitation only because they are simpler in handling and thus are more suitable for workflow automation (25, 26). Nevertheless, precaution needs to be taken to avoid precipitation of lipid classes like TG or cholesteryl esters (unpublished observation).

While the extraction of fluidic samples is straightforward, solid samples like tissues need homogenization prior to extraction. This step needs careful evaluation since lipid recovery could be compromised by various factors, including sample concentration, solvent, and the method applied for homogenization (27). Thus, inappropriate conditions may lead to a selective loss of either nonpolar or ionic lipid classes. Furthermore, it should be emphasized that each type of sample (e.g., solid vs. fluidic, urine vs. blood, blood plasma vs. serum, etc.) will eventually require dedicated considerations (which are however beyond the scope of this review) (28).

In summary, it should be noted that each extraction protocol has its inherent merits and drawbacks, and none of them fits equally well for all lipid categories and classes, which makes it necessary to choose the protocol best suited for the lipids under investigation and the individual needs of the study (29). Solid phase extraction (SPE) can be a good enrichment step when only a few lipids are of interest in a targeted approach (30) (Fig. 2). SPE is beneficial for further fractionation of total lipid extracts, allowing separation of phospholipids or sphingolipids even into individual classes (31, 32, 33). Another application of SPE in lipidomics is the selective enrichment of low-abundance lipids like steroids (34), *N*-acyl phosphatidylethanolamines (35), long-chain base phosphates (36), or glycosphingolipids (37). If necessary, further analytical enhancement of individual lipid classes is possible by derivatization, which results in an enhancement of ionization efficiency and/or introduction of characteristic fragments (38, 39, 40) (Fig. 2). It is advised that whatever method is used for lipid sample preparation, it is always better to add internal standards (ISs) prior extraction to the samples for internal control and quantification (41).

## Direct infusion—MS

**Fig. 3:**
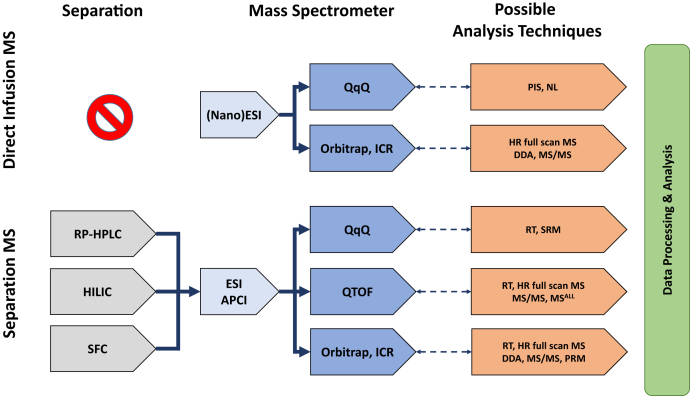
Frequently used instrumental setups. This figure shows an overview of frequently used combinations of chromatography, MS (ion sources and analyzers), and the possible associated analytical techniques, which are provided by the corresponding instrumental setup.

In the early days of lipidomic analysis, direct infusion systems coupled to a triple quadrupole (QqQ) mass spectrometer were the state of the art (Fig. 3). Worth mentioning in this respect are the instrumental settings proposed by the groups of Han and Gross (3, 7) and Liebisch *et al.* (14, 42). The former used a setup relying on either a syringe pump or a static nanoESI source (Nanomate), whereas the latter used a HPLC-based flow injection system. In such a setting, the QqQ is typically utilized for a combination of precursor ion and neutral loss scans. This method is particularly useful if characteristic head group fragments or neutral losses are available, like it is the case for most phospholipids (43, 44, 45, 46, 47) and sphingolipids (48, 49, 50, 51). In addition, precursor ion or neutral loss scans on specific fatty acyl fragments/neutral losses complement the overall picture of a given lipid structure. If all these pieces of information are properly put together, it is possible to identify not only the lipid class and the cumulative number of fatty acyl carbons and double bonds by its corresponding head group fragmentation (e.g., phosphatidylcholine [PC] 38:4) but eventually also the constituent fatty acyls by their specific scans (e.g., PC 18:0_20:4) (7). Since any chromatographic information and separation are missing, it is good advice to include as much fragment information as possible for increasing the selectivity of the analytical system such as multidimensional MS (52). For improved selectivity, alternatively, contemporary direct infusion platforms use high-resolution mass spectrometers, with the gold standard nowadays being Orbitrap MS (53), often coupled to a Nanomate nanoESI chip (Fig. 3) (54, 55, 56). Achieving a mass accuracy of better than 3 ppm enables this mass analyzer to pin down the elemental composition of precursor and fragment masses alike (57, 58). The idea of this setup is the generation of full scan spectra of intact lipid adduct ions and MS/MS spectra of each detected lipid species, both at a mass resolution of 100,000 or higher (54, 55, 59, 60, 61). Furthermore, the nanoESI chip offers another set of advantages compared with conventional direct infusion systems: *1*) It relies on one nanoESI needle per sample, and therefore, any carryover from the injection system is abolished (62). *2*) NanoESI significantly enhances signal intensity resulting in improved limits of detection (63). Generally, direct infusion MS methods benefit from a steady ionization environment, generating very robust quantitative data. Consequently, just one or eventually two nonendogenous ISs for each lipid class are sufficient for quantitation, which is in sharp contrast to the requirements concerning the number of IS for lipid species separation-based methods (7, 41, 53, 62). Nevertheless, it is important to strictly control the concentration of the infused lipids because at concentrations above 10 μM, aggregation of lipids starts to distort quantitative data (4, 64), and at above 100 μM (multiply charged) multimers (e.g., [3M + 2Na]^2+^ or [2M + 3Na]^+^) start to be the dominant forms (3). Furthermore, it is important for quantitation to allow the formation of only one adduct, preferably the one with maximal ionization efficiency. This is the reason why ammonia salts are often used, because they result in PC and SM to be detected mainly as protonated compounds (4), they increase the ionization efficiency of TG as NH_4_^+^ adducts and anionic lipid classes, such as phosphatidylglycerol, phosphatidylinositol, phosphatidylserine, phosphatidic acid, and cardiolipin as deprotonated species (63). Another issue, which could hamper quantitation, would be multiply charged compounds, distributing the intact lipids intensity over several charge states. Since lipids are rather small molecules with a mass below 1,000 Da, this phenomenon is just observed for cardiolipin, phosphatidylinositol phosphate, or gangliosides, which are not the bulk lipid classes. In these cases, the existence of several charge states has of course to be factored in for quantitative aspects. The drawback of direct infusion MS methods is the inherently limited ionization capacity of electrospray leading to ion suppression effects, which results in detection problems for minor lipid constituents of certain nonpolar lipid classes when, for example, compared with ultrahigh-performance supercritical fluid chromatography (UHPSFC) (9). This physical limitation could be overcome either by the use of chromatography or by selective enhancement/suppression techniques, where ionization of specific lipid classes is enhanced by the addition of certain additives, for example, NH_4_OH or LiOH (63, 65). Another drawback of any direct infusion MS approach is the lack of chromatographic information (retention time [RT]) for identification. This can be a particular challenge with lipid extracts rich in overlapping isomeric and isobaric species because isomers and isobars result in mixed MS/MS spectra and tend to complicate data interpretation, although strategies to overcome this issue have been proposed recently (60).

## Separation—MS

### Chromatography

By enabling molecular separation, chromatography opens up another analytical dimension for the analysis of lipids by MS (Fig. 3). On the one hand, the separation of total lipid extracts reduces the complexity of mass spectra, whereas on the other hand, RT provides valuable information about the lipid identity. On the downside, chromatography often needs more demanding and sophisticated quantitation strategies, when compared with direct infusion MS. Reverse-phase (RP)-HPLC, the most widely used separation technique in lipidomics, separates lipid species by their hydrophobic fatty acyl moieties according to the equivalent carbon number model (66). When coupled to QqQ, one specific mass transition in selected reaction monitoring (SRM) for each lipid species is essential for unambiguous identification and further quantitation. This is even possible in a validated clinical setting as has been shown for the analysis of ceramides (67). When using the Orbitrap option for such a platform, molecular species are underpinned by high mass accuracy. Depending on the instrument type and availability of parallel and/or sequential acquisition modes, full MS/MS spectra are either obtained in high or in nominal mass resolution (68). When using a Q-Exactive, parallel reaction monitoring, a product ion scan of a precursor mass selected in Q1 and fragmented in the higher-energy collision dissociation cell, can be used to increase the duty cycle when compared with SRM on this kind of instrument (69, 70). Furthermore, the use of a nanoHPLC system could even further increase the coverage by a factor of three (69). In contrast to RP-HPLC, hydrophilic interaction liquid chromatography (HILIC) separates lipids according to their polar head group into lipid classes. Because of the coelution of all molecular species of a lipid class in a very narrow RT window, this kind of chromatography is very convenient for quantitation, because similarly to shotgun lipidomics, one IS per lipid class could be considered (71). HILIC is often used for the analysis of polar phosphorylated lipids (e.g., LPA, S1P, and bis(monoacylglycerol)phosphate), either coupled with QqQ in SRM mode (15, 72, 73), with an Fourier transform-ion cyclotron resonance-MS (ICR-MS) in all ion fragmentation mode (74), with a Q-Exactive in data-dependent MS/MS mode (75), or to an Orbitrap generating high-resolution full scan spectra and nominal resolution linear ion trap MS/MS spectra in parallel (24). In a HILIC/Orbitrap MS setup, it was even possible to selectively monitor the ^13^C labeling enrichment status of the glycerol backbones of all individual lipid species of a lipid class within just one cumulative MS/MS spectrum per lipid class (76). The merits of HILIC separation running on an ultrahigh-performance liquid chromatography (UHPLC)/quadrupole TOF (QTOF)-MS system in high-resolution full scan MS/MS mode have recently resulted in the highest number of gangliosides being reported so far in one chromatographic run (37). The big advantage of this system is its high duty cycle caused by the speed of both the QTOF and the UHPLC. UHPSFC has recently become a very promising separation technique. It excels by its high chromatographic resolution and its speed. The coupling of UHPSFC running in HILIC-like mode to a QTOF mass spectrometer resulted, for example, in separation of 25 lipid classes in just 6 min runtime (77). When compared with UHPLC, it became evident that UHPSFC has the potential to increase the coverage of the lipidome by a factor of 3.4 with a decrease of analysis time by 40% (9).

### Ion mobility spectrometry

Ion mobility spectrometry (IMS) separations coupled with MS (IMS-MS) have recently been added to many lipidomics workflows to investigate and separate isomeric and isobaric lipid classes and species. IMS enables rapid gas phase-based size separations by employing different electric field and buffer gas introduction combinations. IMS-based techniques include field asymmetric waveform ion mobility spectrometry (FAIMS), drift tube ion mobility spectrometry (DTIMS), traveling wave ion mobility spectrometry (TWIMS), and trapped ion mobility spectrometry (TIMS) (78, 79). FAIMS (or differential ion mobility spectrometry) is one of the most commonly used IMS-based techniques in current lipidomic studies since its baseline separates lipids of different classes in <1 s (80) and provides trend line groupings (81). DTIMS, TWIMS, and TIMS are also widely used in lipidomic analyses since they can be easily coupled with LC separations. The drift time versus *m/z* plots for these separations illustrate trend lines for each lipid class similar to the FAIMS results (82, 83, 84, 85). Moreover, it has been shown that DTIMS with a 4 Torr and 1 m IMS drift region separated lipid isomers such as *sn-1/sn-2* positional, *cis*/*trans* double bond and stereochemical isomers (e.g., R vs. S) (82). While these were very promising results for standards, many of the isomers were only partially separated, limiting the utility of this DTIMS platform for full lipid species separations in complex mixtures and sample types. To obtain better DTIMS separations, both the pressure and length of drift tubes have been increased and advanced multiplexing techniques employed, illustrating greatly improved lipid separation (86, 87). Advances in the other IMS techniques are also showing exciting lipid separation enhancements including long trapping times in TIMS (88), FAIMS devices constructed with higher voltages than commercial versions (89), and the structures for lossless ion manipulation TWIMS platform having a compact serpentine ion drift path ranging from 16 to 500 m (90, 91), and enabling ultrahigh resolving power IMS separations (92, 93).

## Lipid identification

**Fig. 4:**
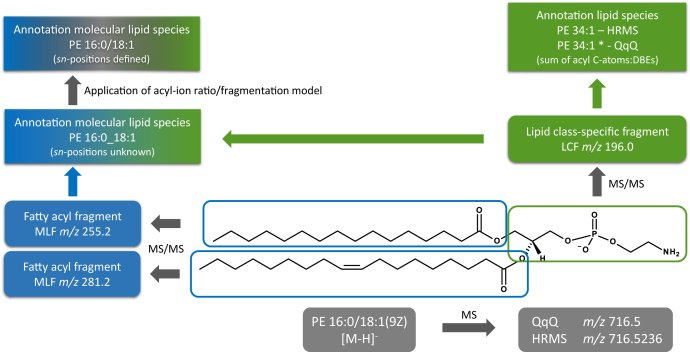
Hierarchical lipid annotation. Annotation levels of PE 16:0/18:1(9Z) are related to mass spectrometric data. Annotation at lipid species level requires precursor mass and LCF ion: Without separation of isobaric PE O-35:1 ([M − H]^−^*m/z* 716.5600) by HRMS, annotation of PE 34:1 ([M − H]^−^*m/z* 716.5236) is based on (∗) the assumption that only even carbon number fatty acyls are present. Molecular lipid species annotation needs the LCF and both molecular lipid fragments (molecular lipid species-specific fragment, here fatty acyl product ions) but does not specify *sn*-positions PE of 16:0_18:1. Annotation at *sn*-position level PE 16:0/18:1 demands further data, for example, the application of fatty acyl ion ratios (98, 99) or fragmentation models (60).

A major challenge in lipidomics is the correct identification of lipid species. Misidentifications were discussed for several studies and represent an ongoing issue (10, 94, 95, 96). Identification and annotation of lipid molecules is based on a hierarchical concept related to the structural details provided by the analysis. Annotation of lipid molecules should mirror the structural resolution, as supported by MS-derived evidence, and follow the recently updated shorthand notation for MS-derived lipid structures (13). The fundamental principle for most lipid classes is the detection of lipid class-specific fragments (LCFs) (see https://lipidomics-standards-initiative.org/resources/lipid-class-specific-fragments) and ions specific for variable components, typically hydrocarbon chains, the so-called molecular lipid species-specific fragments (see https://lipidomics-standards-initiative.org/resources/lipid-molecular-species-fragments) (97) (Fig. 4). Consequently, the detection of only LCF justifies the annotation of lipid species, that is, the sum composition of carbon atoms and double bond equivalents of the hydrocarbon chains. Additional detection of molecular lipid species-specific fragments approves the annotation of hydrocarbon chains, that is, the annotation of molecular lipid species. The determination of *sn* positions requires additional data, for example, application of fatty acyl chain ratios either experimentally determined for specific molecules (98) or included into algorithms (99) as well as response models (60). The identification of double bond positions or other structural details requires sophisticated methods like ozone-mediated (100) or radical-mediated (101) cleavage, derivatization (102), gas-phase chemistry (103), or charge remote fragmentations (104, 105, 106) to generate structure-specific ions.

**Fig. 5:**
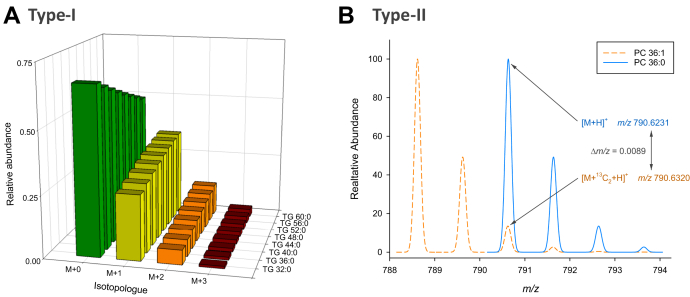
Isotopic effects. A: Type I isotopic effect illustrated for triacylglycerols with increasing number of carbon atoms in all fatty acyl chains. The fraction (abundance is normalized to sum of all isotopologues) of M + 0 decreases, and higher isotopologues increase with the number of carbon atoms. B: Type II isotopic overlap occurs in DB series, that is, within a lipid class, the M + 2-isotopologue of an unsaturated species overlaps M + 0 of species with one double bond less. The overlap of PC 36:1 with PC 36:0 is shown (isotopologue abundance is normalized to the monoisotopic peak M + 0).

Another important aspect to achieve correct identification is the consideration of isomeric (see https://lipidomics-standards-initiative.org/resources/isomeric-overlap) and isobaric overlap (see https://lipidomics-standards-initiative.org/resources/isobaric-overlap). Thus, PC 33:1 is isomeric with PE 36:1, that is, their sum formula is identical. Therefore, in the positive ion mode, [M + H]^+^ or [M + Na]^+^ ions of these species have identical *m/z*, which requires an additional analytical dimension like MS/MS and formation of distinct LCFs for separation. Isobaric overlap may occur from variations in bond types, for example, ether bond containing PE O-35:1 ([M − H]^−^ *m/z* 716.5600) has the same nominal *m/z* as diacyl PE 34:1 ([M − H]^−^ *m/z* 716.5236). Such kind of isobaric overlap may require assumptions based on biological knowledge, like the presence of only even carbon number fatty acyls (Fig. 4), to annotate species when high-resolution MS (HRMS) is not available. Another isobaric interference inherent in lipidomes is the so-called type II overlap (107) occurring in double bond series within the lipid class (Fig. 5B). This interference on monoisotopic peaks results from the second isotopic peak of a species with one additional double bond, for example, [PC 32:1 + H + ^13^C_2_]^+^ overlaps with [PC 32:0 + H]^+^). These isobaric peaks only have a mass difference of 8.94 mDa. Therefore, when confident separation of these species is not ensured, type II overlap needs to be evaluated. In nominal resolution MS (low-resolution MS), this effect is typically corrected by calculating isotope patterns in both direct infusion (107, 108) and lipid class separation (72). In contrast, sufficient resolving power in HRMS (e.g., Orbitrap or ICR) can resolve this isobaric overlap (54). Finally, in-source fragmentation could lead to misidentification (see https://lipidomics-standards-initiative.org/resources/in-source-fragmentation) and should be evaluated when ion source parameters are optimized (109, 110).

In general, the identification principles discussed previously apply to all types of lipidomics approaches. When additional separations, such as chromatography, are utilized, this extra dimension can reduce isomeric and isobaric overlaps. Here, the RT should be used as an additional criterion for lipid species identification. Thus, for lipid class separation like HILIC, the observed RT is similar for the respective lipid class and follows polarity shifts, for example, by hydrocarbon chain length (37, 77) or introduction of additional hydroxyl groups (111). In RP-HPLC, the separation is based on the hydrophobic interaction, which is related to the length of hydrocarbon chains, the number of double bonds, and other structural features, such as polar functional groups. These structural elements directly relate to the RT of the lipid molecules and therefore could be used in mathematical models to justify the correct lipid molecule annotation (66, 112).

## Quantification of lipids

The lipid composition of cell membranes or organelles defines their biophysical properties and function (1, 113). Only an accurate quantification of lipid classes and their species as a basis for lipid composition will permit a detailed understanding of the functional role of lipid species. Moreover, repositories of lipid species for any kind of biological material are only valuable in molar concentrations but not in arbitrary units. Molar concentrations make the determination of mass or ratio changes possible and meaningful for studying lipid metabolism. Finally, the confirmation and validation of lipid biomarkers in different laboratories using concentrations is straightforward and will speed up the transition of such markers into clinical diagnostics (10).

The vast majority of lipidomic methods is based on ESI. It is a fundamental principle of this type of ionization that solvent composition, additives, and other components influence the ionization efficiency (52, 64). This influence, also termed matrix effect, could be compensated by addition of IS exposed to the identical ionization conditions (41). Accurate compensation will only be possible if the molecule used as IS is chemically similar (preferably identical) to the analyte and coionizes with the target molecule (114). Therefore, at least one exogenous IS should be added per lipid class as a minimum requirement for reliable comparison.

The classical way for accurate quantification by application of calibration curves using authentic standards and matching stable isotope-labeled IS is only feasible for targeted analysis of a few analytes (67). In lipidomics, structure-response relationships within each lipid class do vary to a certain extent, with some lipid classes being predominantly dependent on the polar head group, whereas others are rather affected by their fatty acyl composition. It is well known that some lipid classes like PC and SM, which are analyzed in positive ion mode using fragment ion *m/z* 184, are slightly affected by structural modifications, double bond content, and fatty acyl chains because of their inherent head group charge. Some lipid classes like nonpolar cholesteryl ester are substantially affected by the number of carbon atoms and particular double bonds in their fatty acyl chains (53). When fatty acyl fragments are used for glycerophospholipid quantification, beside the number and position of double bonds, carbon number and also the *sn* positions determine their responses (60). These effects can be addressed by the application of response factor models (53, 60). One factor pertaining to all lipid classes is the so-called type I isotopic effect (107) that describes the decrease of the proportion of monoisotopic peaks for increasing number of carbon atoms (Fig. 5A). This effect could be simply corrected based on the calculated isotopic pattern (107). As discussed previously for identification, when type II overlap is present, it needs to be corrected also for accurate quantification (Fig. 5B). Substantial overlap may occur, for example, in nominal resolution direct infusion analysis when PC and SM M + 1 isotopologues overlap with monoisotopic peaks in positive ion mode precursor ion scans of *m/z* 184 (108). In HRMS using Orbitrap or ICR-MS, the partial overlap of type II isobaric peaks may affect accurate quantification because of peak interference. This phenomenon requires evaluation and resolution-dependent data analysis (115).

Besides appropriate data handling, other factors like background signal or preanalytic conditions could substantially influence lipid molecule concentrations as discussed previously. Too high or even false reporting may originate from a chemical background. For example, saturated fatty acids like palmitic and stearic acid are ubiquitous in substantial concentrations (116), and therefore, blank samples should monitor such background especially when a limited amount of sample is subjected to analysis.

## Data reporting

So far, most of the published lipidomics studies report lipid lists using several dialects of lipid shorthand names (117) and custom formatting of lipid quantities and study variables or other proprietary formats. However, data in such semistructured formats may not be portable because of number encoding issues, and it may be inaccessible to computational approaches because of the absence of an underlying scheme describing the structure of the data. Data repositories such as MetaboLights (118) already require the deposition of both raw data and experimental metadata in structured and accessible formats to allow for better reproducibility and transparency of experimental results. However, for MS data from small molecules in general and lipids in particular, additional information that provides normalized and unambiguous shorthand lipid names, appropriate to the level of structural resolution, as well as details on the *m/z* signals used for identification and quantification in addition to preanalytical study parameters such as RT and CCS values, is vital to support reported identifications and quantities. This additional evidence facilitates crosschecking for unfounded identifications and typical analytical or data analysis issues (see aforementioned). The data format mzTab-M for metabolomics addresses these issues (119). As a tab-delimited and spreadsheet-like format, mzTab-M is easily accessible by both humans and machines, based on a well-defined pattern and encoding. It captures experimental metadata, summary information on small molecules across assays, MS features as a basis for quantitation, and evidence to support reporting of individual lipid or feature group identifications. The format was developed with widespread consultation of different approaches taken in the field and involvement of software teams from academic research groups as well as industry. The standard has undergone a rigorous peer-review process by both the Metabolomics Standardization Initiative and the Human Proteome Organization's Proteomics Standards Initiative to ensure that the resulting specification is of high quality and mature. In addition, a web application for conveniently validating mzTab-M files by a graphical user interface and a command line validator are available (120). Support for mzTab-M as an output format has already been included in LDA 2 (121), MS-DIAL 4 (122) and MZmine 3 (unpublished), whereas support for mzTab-M as an input format is available in GNPS (123) and MetaboAnalyst (124).

## Conclusion

Good lipidomic practice should consider the entire lipid workflow starting from sample collection, lipid extraction, mass spectrometric analysis, identification, and quantification of lipid molecules (Fig. 1). To ensure data quality, the workflow should be evaluated before its application. The depth of method validation could be limited when research questions are addressed based on semiquantitative data, but clinical studies or biomarker validation require quantitative data generated with fully validated methods. As a guidance for researchers and reviewers, LSI currently refines the guidelines for lipidomics including a checklist for quality reporting (10). Other currently ongoing projects should further support and promote the development of lipocentric hierarchical databases like LIPID MAPS (125), SwissLipids (http://www.swisslipids.org/#/) (126), comprehensive lipid compendia, and literature collections, like ”The LipidWeb” (https://www.lipidmaps.org/resources/lipidweb/lipidweb_html/index.html) and the introduction of mzTab-M as a small molecule MS transcommunity data format (119). The aim of these developments is to improve the quality of reported lipidomics data to take lipidomics research to the next level, that is, to facilitate a better understanding of lipid molecule functions in health and disease.

## Data availability

All data are contained within the article.

## Conflict of interest

The authors declare that they have no conflicts of interest with the contents of this article.
